# Silent Struggles: Navigating Secondary Traumatic Stress in Dutch Practitioners Working With Individuals Who Have Committed Sexual Violence

**DOI:** 10.1097/JFN.0000000000000579

**Published:** 2025-11-03

**Authors:** Marije Keulen-de Vos, Funda Sanci

**Affiliations:** 1 de Rooyse Wissel, Oostrum, The Netherlands; 2 Faculty of Psychology and Neuroscience, Maastricht University, Maastricht, The Netherlands

**Keywords:** Coping, forensic nurses, secondary traumatic stress, sexual offense histories, social support

## Abstract

**Background:**

Exposure to stories of sexual violence can lead to secondary traumatic stress (STS) or the occurrence of posttraumatic stress symptoms in forensic nurses and other professionals.

**Aims:**

To investigate the prevalence of STS among Dutch nurses and other professionals involved in the treatment of individuals convicted of sex offenses, and the predictive impact of coping styles and social support on STS levels.

**Method:**

Using a cross-sectional survey design, we collected data from 37 nurses and 66 other practitioners.

**Results:**

Our findings indicate a low prevalence of STS symptoms among the participants. Avoidant and active coping were associated with STS, but social support was not.

**Conclusion:**

The relationship between social support and STS may be more complex than previously thought and warrants further research. Targeted interventions are needed to support the development of effective coping mechanisms that have the potential to mitigate the impact of STS.

Key points

- Secondary, or vicarious, trauma occurs in forensic nurses and should be recognized and addressed.
- Nurses struggling with the effects of secondary trauma might experience symptoms such as emotional exhaustion, intrusive thoughts, and heightened levels of anxiety
- Healthy coping strategies, including proactive problem-solving, seeking emotional and informational support, are important to mitigating the effects of secondary trauma.

Sometimes, forensic nurses are exposed to the challenging task of delving into the complex psychosocial dynamics underlying sexual violence. For example, nurses are exposed to aversive details related to client histories. They read and listen to graphic descriptions of sexually violent events, and listen to the clients’ explanations or describe their lived experiences. The continuous exposure to the narratives of sexual violence can significantly impact the mental and emotional well-being of forensic nurses and other practitioners, leading to the development of secondary traumatic stress (STS). STS has been defined as the occurrence of posttraumatic stress symptoms following indirect exposure to traumatic events via a close personal or professional relationship with one or more persons (Bride & Kintzle, 2011). Nurses may experience symptoms such as emotional exhaustion, intrusive thoughts, and heightened levels of anxiety (Frost & Scott, 2022). Addressing the impact of sexual violence on practitioners who are involved in the treatment and supervision of individuals who are convicted of sexual violence is paramount, necessitating robust support systems and self-care strategies.

## Risk and Protective Factors for STS

Several factors can predict the development of STS in forensic nurses and other practitioners. For example, personal characteristics such as high levels of empathy and sensitivity may increase susceptibility (Pirelli et al., 2020). High levels of empathy, sensitivity, and emotional openness can enhance an individual’s ability to connect with clients on a deep level, but they also increase susceptibility to absorbing and internalizing the traumatic experiences of others. Poor self-care practices, including inadequate boundaries between work and personal life, can exacerbate the risk (Frost & Scott, 2022). Without adequate active self-care practices such as regular breaks, exercise, seeking support from colleagues and friends, supervision, and engaging in enjoyable activities outside of work, nurses and other practitioners may find it increasingly challenging to manage the emotional toll of their work (Pellegrini et al., 2022).

Organizational factors, such as high caseloads and limited resources for coping, also contribute significantly to the likelihood of experiencing STS (Bell et al., 2019). For example, high caseloads increase the workload, leaving professionals with limited time to process their own emotional responses and adequately recover between sessions (Quinn et al., 2019). In addition, a lack of resources, such as access to supervision, peer support, and professional development opportunities, deprives nurses and therapists of essential tools and strategies needed to manage their stress effectively (Sutton et al., 2022).

Coping strategies can act as a buffer against the adverse effects of secondary trauma (Folkman & Moskowitz, 2004). Nurses who use healthy active coping mechanisms, such as regular self-care practices, supervision, and ongoing professional development, are better equipped to manage the emotional toll of their work (Pellegrini et al., 2022). Active coping involves proactively addressing stressors through problem-solving, seeking support, and using effective strategies to manage emotional distress (Gerich & Lehner, 2023). On the contrary, avoidant coping styles such as denial, substance use, and avoidant behavior may heighten vulnerability to STS.

Social support, encompassing emotional, informational (e.g., advice), and instrumental (e.g., practical help) support from colleagues, friends, and family, also plays an important role in dealing with STS symptoms (Rzeszutek et al., 2015). Robust social support networks act as a protective factor, providing a mechanism for individuals to share their experiences, seek guidance, and receive empathy. For example, Velando-Soriano et al. (2020) examined social support in the context of nursing. Social support received by nurses in the workplace from supervisors and coworkers was found to play a fundamental role in preventing mental exhaustion.

Recognizing predictive or potentially protective factors is crucial for understanding STS in a forensic mental health context. Understanding these predictors helps in tailoring training programs and providing targeted resources that can bolster practitioners’ resilience and coping mechanisms. Ultimately, being mindful of STS not only contributes to the well-being of professionals and staff retention but also contributes to the overall resilience of the forensic mental health system.

## The Current Study

This study was conducted to add context-specific insights to what is known, highlighting how STS manifests in the particular group of forensic nurses and other professionals involved in sex offender treatment. The study aims were to examine (1) the extent to which STS occurs among forensic practitioners in the Netherlands who are involved in the treatment and supervision of sex offenders, and (2) whether STS is influenced by coping and social support. Specifically, we examined whether avoidant or active (approaching) coping is predictive for levels of STS in Dutch professionals. We expected that use of an avoidant coping style was predictive for higher levels of STS because it prevents practitioners from effectively processing and managing the distressing emotions and experiences associated with their work. Next, we expected that an active coping style was predictive for lower levels of STS because it involves direct engagement with the stressors and seeking constructive solutions. Furthermore, we examined whether the level of STS was predicted by social support of significant others, family or friends. We expected that more social support of these three social groups was predictive for lower levels of STS.

## Methods

### Study Design and Participants

This study used a cross-sectional survey design. The target population consisted of practitioners, including forensic nurses, working in the field of forensic psychiatry in the Netherlands, who were 18 years or older and engaged in the treatment or supervision of clients with sexual offense histories.

### Instruments

With the permission of the original authors, the Secondary Traumatic Stress Scale (STSS) and Brief COPE were translated into Dutch through a typical back-translation procedure. The process began with an initial translation of the material from the source language (English) into the target language (Dutch) by the second author. Next, the first author who is fluent in both languages but was unaware of the original content—translated the items back into the source language (English). In a consensus meeting between the authors, this “back-translation” was compared with the original source text to identify any discrepancies, misunderstandings, or loss of meaning. No changes were made to the translated items.

#### Secondary Traumatic Stress

We used the STSS (Bride et al., 2004), which is a self-report instrument designed to assess the frequency of intrusion, avoidance, and arousal symptoms associated with secondary traumatic stress. The STSS consists of 17 items that are coded on a 5-point Likert-type rating scale (1=*never*; 5=*very often*) and divided across three subscales: intrusion, avoidance, and arousal. A higher score reflects greater frequency of secondary trauma symptoms. The total score can range from 17 to 85. Several studies show adequate to good reliability (Cronbach’s alpha levels >.80) and validity in the English and French languages (Benuto et al., 2021; Jacobs et al., 2019; Manning-Jones et al., 2016). In our study, the internal consistency coefficients (Cronbach’s alpha) were .58 for the intrusion scale, .72 for the avoidance scale, and .84 for the arousal scale. The Cronbach’s alpha for the STSS total score was .89.

#### Coping

We used the Brief COPE (Carver, 1997), which consists of 28 items that measure coping responses in response to difficult or stressful situations. Items are scored on a 4-point Likert-type scale (1=*I did not do this at all*; 4=*I did this a lot*). Scores are presented for the two overarching coping styles: avoidant coping and approach coping. Avoidant coping, which is characterized by the subscales of denial, substance use, venting, behavioral disengagement, self-distraction, and self-blame. Approach coping is characterized by the subscales of active coping, positive reframing, planning, acceptance, seeking emotional support, and seeking informational support. The higher the scale score, the more use of a specific coping strategy. The range of the total avoidant and active coping subscales was 12–48. Several studies show adequate to good reliability (e.g., alpha levels > .80) and validity in the Chinese and English languages (Huang et al., 2020; Solberg et al., 2022). In our study, the Cronbach's alpha were .71 for avoidant coping and .86 for approach coping.

#### Social Support

We used the Dutch version of the Multidimensional Scale of Perceived Social Support ([MSPSS]; De Clerck, 2009) that measures perceived social support on three subscales: significant others, family, and friends. The MSPSS consists of 12 items, rated on a 7-point Likert-type scale (1=*completely disagree,* 7=*completely agree*). Higher scores reflect greater levels of social support. The total score can range from 12 to 84. The MSPSS has been shown to have good internal and test-retest reliability, and good validity in studies across the United States, Europe, and Israel (language not specified in publication; Wang et al., 2018). In our study, the Cronbach's alpha was .88 for the significant others scale, .94 for the family scale, and .94 for the friends' scale.

### Ethical Considerations

The study was reviewed by the institutional review board of the VIGO group, of which de Rooyse Wissel is part (reference ERC/RW-23-003). The board provided administrative permission and scrutinized the ethics of the study. Among other things, the burden placed on participants, any possible negative effects (none were expected), the procedures for data collection, storage, and protecting anonymity, and the measures chosen were considered. Potential participants were first presented with a digital informed consent page providing information about the purpose of the study and the questions that could be expected. Furthermore, it was specified that participants could end their participation at any time and that participation was anonymous.

### Procedures

An online survey was created with SurveyMonkey Inc. (2023). The first section collected background information from the participants (i.e., age, gender, years of working experience in the current specialty, job description). The next sections consisted of questions pertaining to the STSS, Brief COPE, and MSPSS. The survey consisted of 61 items in total. The survey was distributed through LinkedIn, given the frequent use of this platform by Dutch professionals. In our advertisements, we included a statement about who could participate and whom they could contact in case the survey was upsetting (e.g., the principal investigator, their manager at work, or their health care provider). The survey was active for four months from December 2022 through March 2023, during which calls to participate were repeated twice to ensure sufficient participation.

As an incentive to complete the survey, participants were given the opportunity to enter a lottery to win one of three gift certificates, valued at €15, €25, or €50, from a popular online store in the Netherlands. There were three gift cards distributed (one of each amount). This information was included in the advertisement text. The lottery page was located on a different URL than the actual survey, and the details entered here could not be connected to the answers provided in the survey. The research data, which does not contain any personal information, was stored on a secure server.

### Data Analyses

All analyses were conducted using SPSS version 29. Demographic data were analyzed with descriptive techniques, such as means, frequencies, and percentages. The survey was started 121 times. Of the 121 participants who started the survey, 18 participants accepted the informed consent but did not answer any questions. These subjects were excluded from the analyses, leaving 103 in the final analyses. There was no missing data for the STSS; the Brief COPE data were missing for 6 participants, and the MSPSS data were missing for 9 participants. No respondents were removed from the set. Outliers were individually assessed based on what type of outliers they are and how much they deviate from the final sample. Starting from a difference of 2.5 standard deviations, removal was considered. No data was removed. We used the Mann-Whitney *U* test (given the non-normal distribution) and a χ^2^ test to compare the two groups (forensic nurses vs. other professionals). To test our hypothesis, we conducted Spearman's rho correlation analyses and a hierarchical multiple regression analysis with the STSS total score as the outcome variable. To examine the predictive relationship between the Brief COPE and the MSPSS, and STSS, we controlled for the number of years of experience on the job. In the hierarchical regression model, we entered years of work experience as a predictor in Step 1, the two Brief COPE in Step 2, and the MSPSS sub-scales in Step 3. Because our study variables had a non-normal distribution, as assessed by Shapiro–Wilk’s test (*p*<0.05), we performed bootstrapping. An a priori power analysis (G*Power 3.1.9.7; Faul et al., 2007) estimated a required minimal sample of 98 participants, for a power of 0.80 with α=.05 and a medium effect size (.15) when considering six predictor variables. We chose a medium effect size because a small effect might require an unrealistically large sample size, while a large effect might require too small a sample, increasing the risk of being underpowered if the actual effect size turns out to be smaller. The sample in our study consisted of 103 participants.

## Results

The participants had an average of ∼10 years of experience working in forensic mental health care. Their median age was 38 and 80.6% was female. There were no significant differences between forensic nurses and other professionals regarding age (*U*=1,293, *z*=.499, *p*=0.618) and years of work experience (*U*=1,067, *z*=−.849, *p*=0.396). There were also no significant differences with regard to gender (χ^2^ =2.137, *p*=0.144). About one third (*N*=37; 33%) of the participants worked on a treatment unit as a psychiatric nurse. See Table 1 for demographics.

**TABLE 1:** Demographic Characteristics of the Sample (*N*=103)

	Forensic nurses (*N*=37)	Other professionals (*N*=66)	Overall sample (*N*=103)
	*Mean*	*Mean*	*Mean*
Work experience (years)	9.2	10.6	10.1
Age (years)	39.7	37.8	38.5
	Frequencies, *N* (%)		
Sex			
Male	10 (27)	10 (15.2)	20 (19.4)
Female	27 (73)	56 (84.8)	83 (80.6)
Other	0	0	0
Professional role			
Treatment role outside unit (e.g., psychologist, skills trainer)		34 (33)		
Treatment coordinator		5 (4.9)		
Management		2 (1.9)		
Supportive services		3 (2.9)		
Probation services		19 (18.4)		

The reported level of secondary trauma in our participants was relatively low, with a median STSS total score of 25. The median scores for the STSS intrusion scale and arousal scale were 8 and 9, respectively. The median score for the STSS avoidance scale was 10. There were no significant differences between forensic nurses and other participants for the total score (*U*=1,135, *z*=− .592, *p*=0.554), nor for the intrusion (*U*=1,201, *z*=− .136, *p*=0.892), arousal (*U*=1068, *z*=−1.056, *p*=0.291), and avoidance scale (*U*=11,488, *z*=− .504, *p*=0.614). The mean scores for the Brief COPE and MSPSS subscales are presented in Table 2. There were no significant differences in the Brief COPE and MSPSS scores between forensic nurses and other practitioners.

**TABLE 2:** Mean Scores for the Brief COPE and MSPSS Subscales

	Forensic nurses (*N*=37)	Other professionals (*N*=66)	Overall sample (*N*=103)
Instrument	*Mean*	*Mean*	*Mean*
STSS—intrusion (possible scoring range 5–25)	7.9	8.0	7.9
STSS—avoidance (possible scoring range 5–35)	10.1	10.3	1.2
STSS—arousal (possible scoring range 5–25)	8.4	8.9	8.8
Brief COPE—avoidant coping (possible scoring range 12–48)	23.5	23.0	23.2
Brief COPE—active coping (possible scoring range 12–48)	34.8	34.6	34.7
MSPSS—significant others (possible scoring range 4–48 divided by 4)	6.3	6.3	6.3
MSPSS—family (possible scoring range 4–48 divided by 4)	5.9	5.4	5.6
MSPSS—friends (possible scoring range 4–48 divided by 4)	6.1	6.1	6.1

*Note*. MSPSS, the Multidimensional Scale of Perceived Social Support; STSS, Secondary Traumatic Stress Scale.

Table 3 presents the Spearman correlations between the STSS total score, Brief COPE subscales, and MSPSS subscales. The Brief COPE avoidant coping scale was significantly related to the STSS. There were no significant correlations between Brief COPE active coping, MSPSS significant others, MSPSS by family and MSPSS friends, and the STSS total score. There were no differences between correlations among these factors in forensic nurses and other practitioners.

**TABLE 3:** Spearman Correlation between Coping, Social Support, and Secondary Traumatic Stress

	STSS total scale		
	Rho	*p*	95% CI
Brief COPE—avoidant coping	0.34	<0.01	0.13, 0.52
Brief COPE—active coping	−0.14	0.21	−0.35, 0.09
MSPSS—significant others	−0.18	0.10	−0.39, 0.04
MSPSS—family	−0.11	0.31	−0.33, 0.11
MSPSS—friends	−0.04	0.74	−0.26, 0.19

*Note*. CI, confidence interval; MSPSS, the Multidimensional Scale of Perceived Social Support.

The first regression model predicting the STSS total score based on years of work experience was not significant. The second regression model predicting the STSS total score based on years of work experience, and the Brief COPE avoidant and active coping was significant, explaining 24% of the variance with Brief COPE avoidant coping as a positive predictor and Brief COPE active as a negative predictor. The third model with MSPSS among the three social groups added in step 3 explained 26.2% of the variance. In this model, the Brief COPE avoidant and active coping were the only significant predictors. See Table 4 for details about the models predicting STSS.

**TABLE 4:** Regression Analysis of the Predictive Value of Coping and Social Support on Secondary Traumatic Stress

Step	*F*	*R* ^2^ (Adj)	∆*R* ^2^		*B* (*SE B*)	β	95% CI	*t*
Step 1	0.95	.01 (.00)		*Constant*	28.25 (1.33)		25.61, 30.89	
				Years of work experience	−0.11 (0.11)	−0.11	−0.30, 0.11	−0.97
Step 2	8.02^*^	.24 (.21)	.23	*Constant*	25.33 (4.51)		15.92, 38.34	
				Years of work experience	−0.09 (0.10)	−0.09	−0.28, 0.09	−0.93
				Brief COPE—avoidant coping	0.85 (0.19)	0.52	0.55, 1.20	4.44^*^
				Brief COPE—active coping	−0.48 (0.12)	−0.48	−0.82, −0.22	−3.88^*^
Step 3	4.37^*^	.26 (.20)	.02	*Constant*	33.03 (6.95)		10.70, 50.02	
				Years of work experience	−0.08 (0.10)	−0.08	−0.27, 0.12	−0.86
				Brief COPE—avoidant coping	0.79 (0.19)	0.492	0.41, 1.21	4.03**
				Brief COPE—active coping	−0.42 (0.13)	−0.398	−0.76, −0.13	−3.16^*^
				MSPSS—significant others	−0.63 (0.99)	−0.078	−3.56, 1.95	−0.61
				MSPSS—family	−0.34 (0.54)	−0.07	−1.6, 0.93	−0.63
				MSPSS—friends	−0.47 (0.86)	−0.06	−2.43, 1.42	−0.54

^*^ Significant at *p*<0.01; ** significant at *p*<.001.

*Note*. MSPSS, the Multidimensional Scale of Perceived Social Support.

## Discussion

Our results show that the level of STS in Dutch practitioners is low, which was consistent with Pirelli et al. (2020) and Hatcher and Noakes (2010), who showed that forensic practitioners do not experience high levels of STS compared with general mental health practitioners. However, Newman et al. (2023) reported that about half of the nurses in a forensic facility had moderate STS scores. Despite the high exposure to aggression and violence, several studies show that forensic care practitioners do not experience particularly high levels of STS and exhibit higher levels of job satisfaction than mental health practitioners (e.g., Happell, Martin et al., 2003; Happell, Pinikahana et al., 2003). Happell, Pinikahana et al. (2003) explain this by hypothesizing that forensic practitioners are highly confident in their competence despite caring for a complex group of clients. In addition, it is often assumed that forensic psychiatry demands a unique perspective and demeanor (Clercx et al., 2021).

It is possible that forensic nurses, and other practitioners for that matter, experience relatively little to moderate STS because they can think differently about the severity of patients’ psychopathology and are relatively unaffected by aggression (Dickinson & Wright, 2008). The findings of a qualitative, exploratory study in Australia indicated that many forensic mental health practitioners felt they needed to maintain emotional distance from forensic clients to maintain their own sense of safety and to avoid their secondary traumatic stress (Harris et al., 2015). It is interesting to note that although some practitioners who work with clients with sexual offense histories are negatively affected by their work, a significant number of these practitioners find satisfaction in their work and actually consider it rewarding because of its challenging nature (Bach & Demuth, 2018). Another explanation for our finding may be that the instrument in our study may not include all aspects of trauma. For example, the PTSD criterion “changes in cognitions and mood” is not part of the questionnaire, even though it may be an important factor to assess STS.

Although several studies have shown that the number of years of work experience affects competencies and skills of forensic care practitioners (Clercx et al., 2023; Dorociak et al., 2017), the number of years of employment in our study did not appear to be associated with STS. This is not in line with various studies that generally find a correlation between work experience and STS; usually, lower STS levels are associated with professionals who are more experienced (Meier & Kim, 2022; Starcher & Stolzenerg, 2020). However, there are also studies that suggest no relationship exists (Ivicic & Motta, 2017). So the existing literature on the relationship between work experience and STS among forensic mental health practitioners presents a complex and nuanced picture. This disparity in findings highlights the need for further investigation to clarify the factors that contribute to this variability. Perhaps caseload is more relevant than the years of experience.

Avoidant coping was a positive predictor (more avoidant coping=more stress), whereas active coping was a negative predictor (more active coping=less stress). Forensic nurses and other practitioners using avoidance may distance themselves emotionally from their work, engage in denial about the severity of the trauma they are exposed to, or distract themselves with unrelated activities. This can lead to the accumulation of unresolved emotional responses, which, over time, manifest as symptoms of STS (Mashego et al., 2023). Conversely, active coping, characterized by proactive problem-solving and seeking support, tends to mitigate the impact of STS (Pirelli et al., 2020). By addressing stressors directly and using available resources, practitioners employing active coping strategies are better equipped to manage the emotional demands of their work, thereby reducing the likelihood of experiencing secondary traumatic stress (Mivshek & Schriver, 2022). However, in our study, the explained variance was ∼25% which suggests that other factors are also likely to influence STS.

Social support was not predictive of secondary traumatic stress in our study. First, the intense and specific nature of the exposure inherent in working with clients with sexual offense histories may render general social support less effective, as friends, significant others, and family members might struggle to relate to or fully comprehend the unique stresses involved (Frost & Scott, 2022). Second, organizational culture and workload may influence the relationship between social support and STS; a supportive, well-resourced work environment enhances the protective effects of social support, while high workloads and toxic workplace dynamics can diminish its effectiveness (Adams, 2017). Third, the relation between social support and STS may be dependent on individual resilience. Resilience can be defined as an active process of positive adaptation following an adverse event at work, involving affective, behavioral, and cognitive personal factors and self-regulatory processes. Perhaps practitioners with higher resilience may be better able to use social support to mitigate STS.

### Implications for Forensic Health Care Professionals

Forensic nurses and other practitioners with low levels of secondary traumatic stress are better able to build empathetic and supportive relationships with clients, thereby increasing the effectiveness of forensic care (Chapman et al., 2024). In addition, they may have more energy and resilience to remain committed to the treatment and supervision of clients with sexual offense histories, enhancing continuity of care (Măirean, 2016; Zeng et al., 2023). They are less at risk for burnout, depression, and other negative consequences of prolonged exposure to sex-related information (Ireland et al., 2022), which can contribute to a positive work environment (Mistry et al., 2022).

This study found a predictive relationship between avoidant and active coping, and secondary traumatic stress. Recognizing the benefits of active coping among practitioners working with clients convicted of sexual violence is crucial for developing effective interventions. For example, the use of individual clinical supervision can assist forensic nurses in considering active coping strategies and reflecting on their practice. It is also important for other professionals to recognize that nurses are the only profession in constant close contact with clients who have committed sexual violence, which means that they may be in extra need of supervision. Organizations should provide regular training on active coping strategies, such as mindfulness and reflective practice. Interventions aimed at enhancing active coping behaviors can mitigate the risk of STS, thereby enhancing the overall effectiveness and well-being of practitioners. Future research should be conducted on the course of STS over time, focusing on the factors that have a positive or negative impact on this course, such as regular intervision and supervision.

### Strengths and Limitations

The study uniquely examines STS in Dutch forensic nurses working with individuals convicted of sexual offenses—a population underrepresented in existing research. There are several limitations for interpreting the findings of our study. First, data collection was limited to self-reporting via an online survey. In addition to self-reporting, it may be advisable to obtain information from informants or to use observational or qualitative methods. A second limitation is that there were predominantly women who participated in the survey. Our findings may not be generalizable to male forensic care professionals. Third, our sample was a convenience sample. Since participants are selected based on their availability and ease of access rather than through a randomized or stratified approach, the resulting sample may hamper the generalizability of our findings. A fourth limitation is that the study had a cross-sectional design, which did not allow for evaluating STS beyond one point in time. In future research, it is important to look at similarities and differences among forensic professionals of various gender identities and target groups, and to consider longitudinal studies that can evaluate STS over time.

## Conclusions

The level of STS in Dutch practitioners working with clients with sexual offense histories was relatively low, but further research is warranted. STS was not predicted by social support but was partially explained by avoidant and active coping styles. These results are informative for both treatment professionals and those responsible for policy and training.
